# Facilely synthesized *n*-type conducting polymer with solution processability, ultra-high conductivity and high stability

**DOI:** 10.1093/nsr/nwad011

**Published:** 2023-01-14

**Authors:** Yongfang Li

**Affiliations:** Beijing National Laboratory for Molecular Sciences, CAS Key Laboratory of Organic Solids, Institute of Chemistry, Chinese Academy of Sciences, China

Conducting polymers (CPs) have drawn great attention since the emergence of doped polyacetylene in 1977 [1]. The solution processability is a key property for the application of CPs, but it is challenging for highly conductive CPs without flexible side chains. Cao *et al*. in 1992 proposed ‘counter-ion induced processability’ to achieve the solution-processable conducting polyaniline [2]. Since then, a variety of solution-processable CPs have been developed and commercialized. However, most high-performance CPs exhibit *p*-type properties with hole transport dominating. The *n*-type CPs dominated by electron-transport lag far behind in conductivity and ambient stability, which limits their applications.

To increase the conductivity of the *n*-type CPs, one common strategy is to employ a large rigid conjugate backbone to improve electron transport, but it requires insulating flexible side chains or surfactants to satisfy its solution processability, which in turn decreases the conductivity. Another effective way is to develop high-performance dopants and *n*-doping methods. By synergistic use of the two strategies, the conductivity of solution-processed *n*-type CPs has been boosted to ∼100 S cm^−1^ [3,4]. Nevertheless, the whole process usually requires complex operations, thus limiting large-scale commercialization.

**Figure 1.**
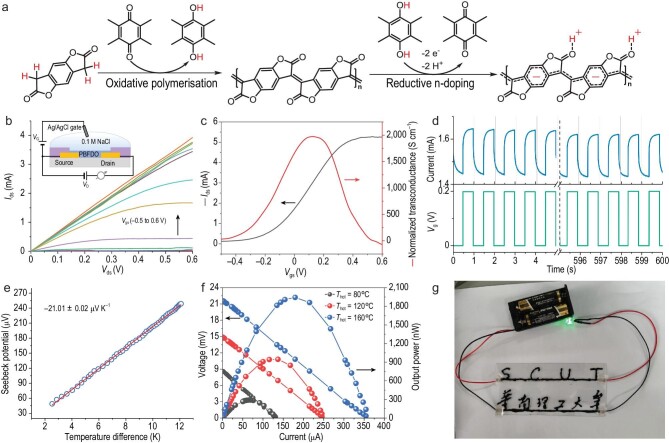
(a) Schematic illustration for the possible mechanisms of combined oxidative polymerization and *n*-doping for the synthesis of PBFDO. (b–d) Performance of the organic electrochemical transistor with PBFDO as the active channel material. (e) Seebeck coefficient fitting of PBFDO at room temperature measured in air. (f) Output voltage and power of the integrated thermoelectric generator. (g) Photograph of PBFDO conductive wires on a polyethylene terephthalate (PET) substrate lighting up an LED. Reproduced with permission [5].

Recently, Prof. Fei Huang *et al.* from South China University of Technology made a breakthrough on the innovation of an *n*-type stable and solution-processable conducting polymer poly(benzodifurandione) (PBFDO) with high conductivity of 2000 S cm^−1^, by a reaction combining oxidative polymerization and reductive *n*-doping (Fig. 1a) [5]. Taking advantage of the reversible redox property of quinone oxidants, they first realized the oxidative dehydrogenation polymerization of benzodifurandione precursor under the existence of tetramethyl-benzoquinone as the oxidant. Then, during the polymerization, the deep-lying reduction energy level of the polymerized conjugated backbone enables the polymer *in situ n*-doped by the generated tetramethylhydroquinone. This process greatly improves the *n*-doping efficiency of the polymer without additional *n*-type dopants and simplifies the operation complexity. Moreover, the strong interaction between the charged conjugated backbone and the solvent results in good solubility and solution processability of PBFDO in dimethyl sulfoxide without the requirement of flexible side chains or surfactants.

They deeply investigated the charge-transport mechanisms of PBFDO by using variable temperature conductivity, Hall effect, magnetoresistance, electron paramagnetic resonance and superconducting quantum interference, and found that conducting PBFDO locates in the critical region of the metal–insulator (M–I) transition and exhibits some properties similar to those of metals such as Pauli paramagnetism and electromagnetic shielding.

Based on the ultra-high conductivity and stability of PBFDO, a variety of organic electronic devices were fabricated (Fig. 1b–g). The organic electrochemical transistor exhibited a high trans-conductance of 1970 S cm^−1^ and excellent operational stability; *n*-type organic thermoelectric devices were prepared, showing stable operation in air with a power factor close to 90 μW m^−1^ K^−2^; flexible electrodes were printed, demonstrating the potential for practical applications in organic electronic devices.

Overall, the success in the synthesis of PBFDO greatly promotes the development of *n*-type CPs and their applications.
